# Supplementary material for: Extending the dynamic range of biomarker quantification through molecular equalization
Source: Nat Commun. 2023 Jul 13;14:4192. doi: 10.1038/s41467-023-39772-z (PMC10344875; doi:10.1038/s41467-023-39772-z)
Supplement: Supplementary file 1 — Supplementary Information [file 41467_2023_39772_MOESM1_ESM.docx]

**SUPPLEMENTARY INFORMATION**

**Supplementary Figure 1 | Effects of non-linear dilution.** Researchers assessing non-linear dilution on the Luminex platform demonstrated that when samples were diluted three-fold, most analytes exhibited a < 3-fold reduction in signal. Only 6% of the tested targets exhibited a proportional 2.75–3.25-fold reduction of signal (demarcated by dashed lines). Data obtained and replotted from Rosenberg-Hasson et al with permission^1^. MFI – mean fluorescence intensity.


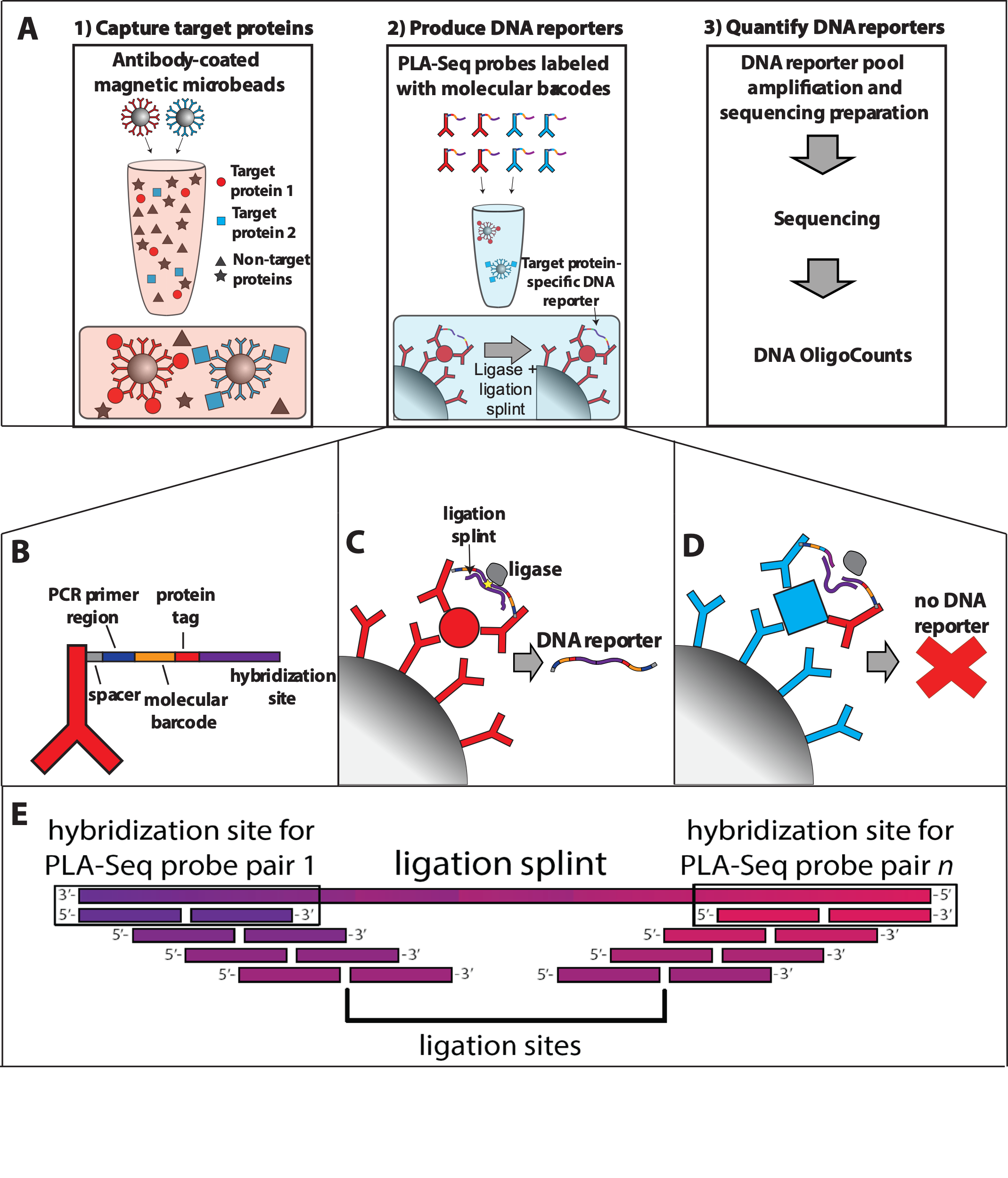
`

**Supplementary Figure 2 | Overview of modified spPLA.** This assay designed is based on work described by Darmanis^2^ and Nong^3^**A)** First, target proteins are captured by capture antibody-coated beads (left). After washing, PLA detection probes with oligo labels are added to the solution to produce a target specific DNA reporter (middle), which is then amplified and prepared for sequencing (right). **B)** Captured targets are labeled with oligo-probe conjugates that have specific protein tags. **C, D)** The right combination of probes must be bound to the target to yield a DNA signaling strand. **E)** The sliding splint approach allows us to design hybridization sites on each dAb’s DNA tag so that dozens or even hundreds of targets can be detected using a single ligation strand. Tiling the hybridization sites along the ligation strand also ensures specificity; if a cross-reactive binding event occurs, the DNA oligos on the antibodies will not line up and no DNA reporter will be produced.

**Supplementary Figure 3 | UMIs enable high precision at low copy number** **but offer low precision at high copy number.** This trade-off makes the tuning of the DNA output concentration (*i.e.*, the number of ligation events) crucial, so that (1) no single target consumes an overwhelming number of reads, and (2) the concentrations aren’t so high that every read has a unique barcode. We determined the desired output DNA concentration to be ~1 fM by sequencing different concentrations of control DNA and observing how the resolution of UMIs changes. The rank-ordered counts of each UMI resulting from different input concentrations of DNA to the PCR reaction can be used to estimate the concentration that confers maximum resolution. As the number of UMIs approaches the read depth of the sequencer, the ability to resolve counts diminishes. The opposite is the case for a small number of barcodes. We observed that the ability to resolve 400 pM vs. 8 pM (red/orange) was worse than 1.6 fM vs. 320 aM (blue/purple). We identified an output of 1 fM DNA as optimal: high enough to minimize Poisson noise but still low enough to be well below the per-target read depth. We note that the optimum DNA output is a function of the sequencer read depth, PhiX fraction, and the PCR reaction volume. Therefore, the optimal DNA reporter output should be verified under other conditions. Triplicates were plotted individually. RPM = reads per million. Source data are provided as a Source Data file.


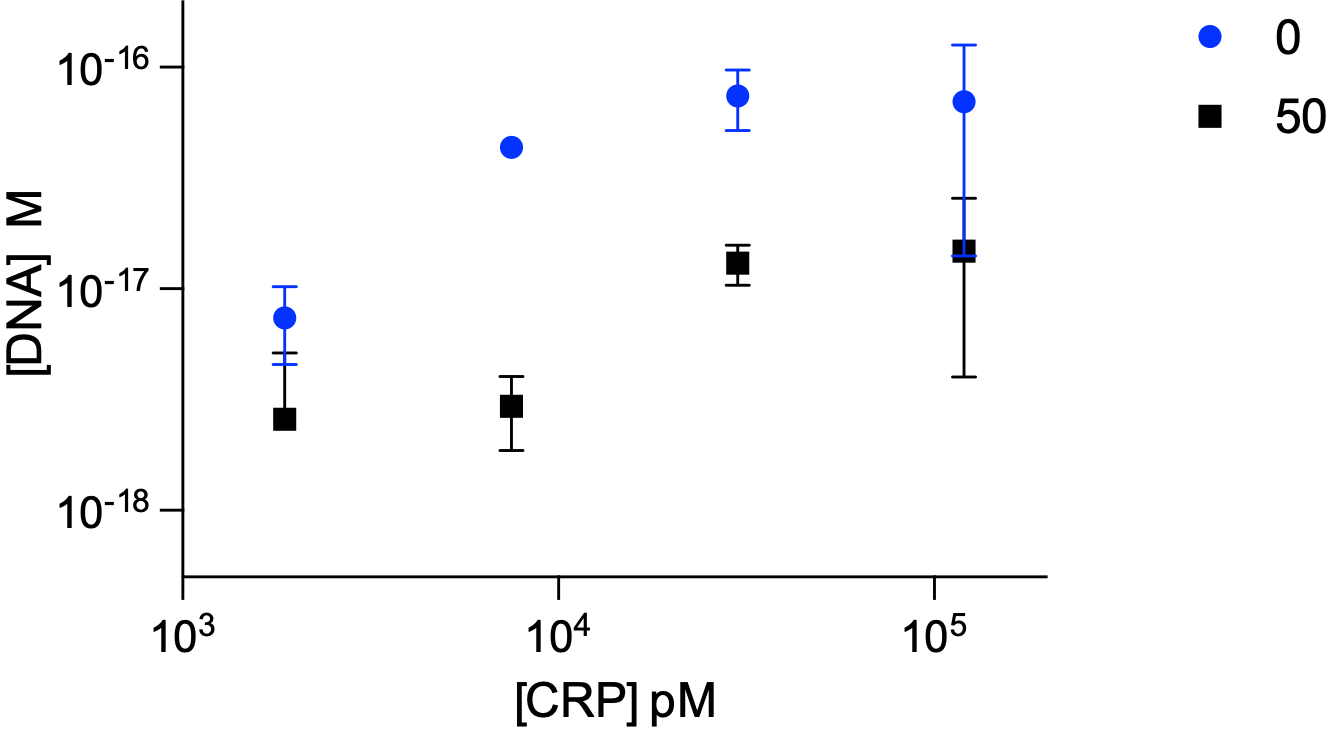


**Supplementary Figure 4 | Signal drop from epitope depletion.**  Binding curves based on qPCR output for CRP using 0.43 pM dAb with 0 or 50 nM of depletant. 50 nM depletant produces a ~10–15-fold drop in signal. N = 3, mean +/- standard deviation. Source data are provided as a Source Data file.

**Supplementary Figure 5 | Background signals increase with probe concentration.** We measured the background signal at each probe concentration in buffer with an HTS readout. From left to right, the four datapoints were from the probe sets for CRP, GDF-15, IL-1ra, and IL-6, respectively. Error bars represent the standard deviation of three replicates from the mean. Source data are provided as a Source Data file.

**Supplementary Figure 6 | Assessing specificity of our EVROS-tuned assay.** We verified the specificity of our assay by demonstrating that the addition of any target, even very high concentrations of CRP, does not appreciably affect the signals from the other targets due to the requirement of two binding events to generate a signal. [CRP] = 121,500 pM, [GDF-15] = 486 pM, [IL-1ra] = 365 pM, and [IL-6] = 1.8 pM. n = 3, mean +/- standard deviation. Error bars represent the standard deviation of three replicates. Source data are provided as a Source Data file.


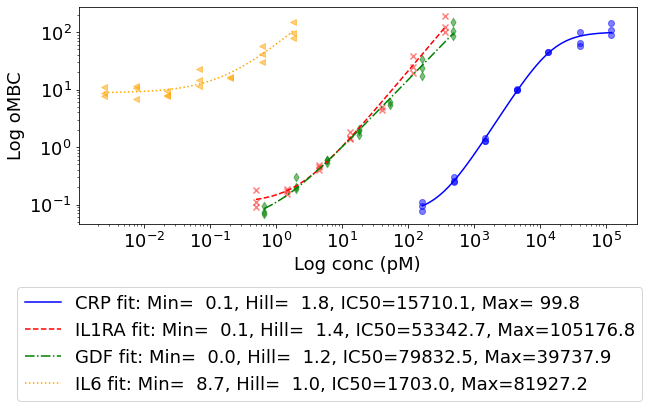


**Supplementary Figure 7 | Four-parameter logistic curve fit for analyte standards spiked in buffer**. The standards described in **Supplementary Table 3** were measured with an EVROS-tuned assay, and we fitted the binding curves as described in the Methods. The determined parameter fits were used to quantify the remaining samples of unknown concentration. Source data are provided as a Source Data file.


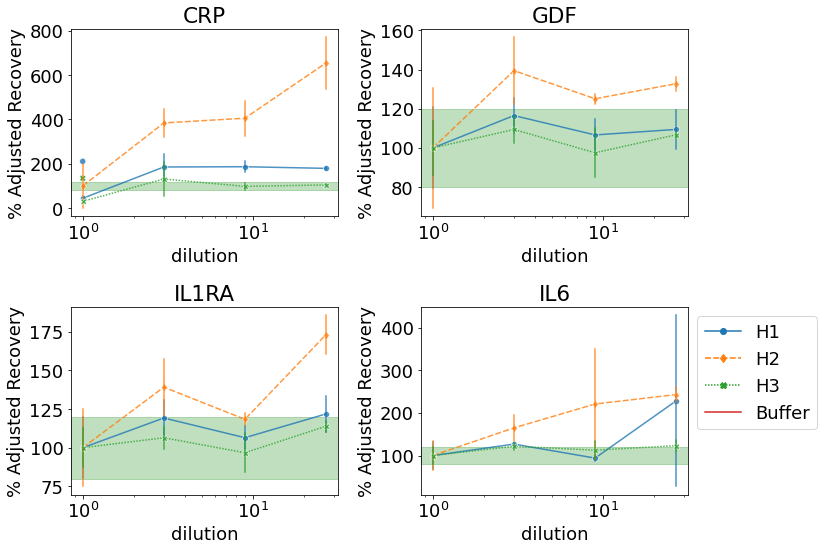


**Supplementary Figure 8 | Non-linear dilution effects observed with spPLA**. A known amount of analyte was spiked into three human serum samples, which were then diluted 3-, 9-, or 27-fold with PLA buffer and measured for analyte recovery efficiency. Each color represents one of three independent human serum samples. The green shaded region demarcates a generous +/- 20% error range for acceptable linearity after dilution. Vertical bars represent one standard deviation for the triplicates (n=3). Source data are provided as a Source Data file.

**Supplementary Note 1 | Derivation of tuning mechanism**

A mechanistic model can be used to predict the tuning behavior of the EVROS system. We applied the following assumptions to equilibrium binding equations to derive our model: uniform K_D_ across the polyclonal pool, K_D_ does not change with increasing number of bound antibodies, any antibody in the pool has an equal likelihood of binding to any epitope on its intended target, and each target has n epitopes where n ≥ 3. We note that these assumptions are too broad to predict behavior exactly but are sufficient to rationalize the trends we observe.

We start with n binding equations describing the equilibrium between *i* bound antibodies and *i+1* bound antibodies,

$K_{D}=\frac{\left[ TA_{i} \right]\left[ A \right]_{tot}f_{Ab}}{\left[ TA_{i+1} \right]}$ for *i* = 0, 1, … , n–1, (1)

where

$f_{Ab}=\frac{\left[ bound dAb \right]}{\left[ total dAb \right]}=1-\frac{\sum_{i=1}^{n} i\left[ TA_{i} \right]}{\left[ A \right]_{tot}}$, (2)

in which $\left[ TA_{i} \right]$ is the concentration of target bound to *i* dAbs, [A]_tot_ is the total concentration of dAbs, and $f_{Ab}$ is the fraction of dAbs bound to target.

In this model, we assumed that all dAbs are from the same species, and then later assigned these to a 3’ or 5’ designation based on probability. In the manuscript, we used equal concentrations of 3’- and 5’-dAbs; however, asymmetric concentrations of dAbs could also be used, and so we have defined f_p_ as the fraction of [dAb] that is the lower concentration. In other words, f_p_ = 0.5 for equivalent dAb concentrations, whereas if the 3’-dAb concentration is twice that of 5’-dAb, f_p_ = 0.33. The resulting signal can be defined using the binomial distribution and summing up the number of targets bound to multiple antibodies times the probability that there is a matching pair of 3’- and 5’-dAbs on each molecule:

$S=\sum_{i=2}^{n} \sum_{k=1}^{i-1} \binom{i}{k}\left[ TA_{i} \right]\left( f_{p}\left( 1-f_{d} \right) \right)^{i-k}\left( \left( 1-f_{p} \right)\left( 1-f_{d} \right) \right)^{k}$, (3)

where f_p_ is the asymmetry of the probes ([limiting probe]/[A]_tot_-[depletant]), f_d_ is the fraction of total antibody that is unlabeled ([depletant]/[A]_tot_), and *n* is the maximum number of binding epitopes on that target. We start summing at i = 2 because fewer dAbs will not produce a signal. We assume that K_D_ is constant for subsequent binding events and is homogeneous (*i.e.,* all Abs in the pool have the same K_D_), which is not necessarily a good assumption for a polyclonal pool but simplifies the math. The impact of this assumption is discussed later. Using Eq.1–3, we can solve for the distributions of the number of bound dAbs to the target molecules.

Increasing the concentration of dAb probes (*[A]_tot_*), or ‘probe loading’, will increase the number of DNA reporters that are produced, which is seemingly an undesired effect. However, this increase is asymmetric with respect to target concentration; the magnitude of this shift depends on [*T*], such that DNA outputs at low target concentrations are affected the least, whereas outputs at high target concentrations are strongly affected. The result is an upward and subtle rightward shift of the response curve as the amount of dAb increases. We can offset this upward shift using ‘epitope depletion’, achieved via the addition of unlabeled antibodies that compete with the DNA-labeled dAbs for the same target epitopes or by adding the two dAbs in different concentrations (i.e., asymmetric probe loading). This results in a fractional decrease in DNA production, shifting the response curve downward in a target concentration-independent fashion, as seen from Eq. 3. The combined effects of probe loading and epitope depletion thus enable precise tuning of the dynamic range of the dose-response curve for each target individually.

For low-abundance proteins, our goal is to move the log-linear range of the signal response curve left or right by changing [A]_tot_. Assuming the dAbs exhibit a constant *K_D_* across binding sites and that the concentration [*T*] of low-abundance proteins is much lower than the *K_D_* of their associated antibodies, the binding curves of these proteins are given by:

$f=\frac{\left[ T \right]}{\left[ T \right]+K_{D}}\approx\frac{\left[ T \right]}{K_{D}}$. (4)

The signal of the assay is proportional to the concentration of target molecules that are bound to two dAbs, $\left[ TA_{2} \right]$, which can be described as a simple linear function of target concentration,

$\left[ TA_{2} \right]\approx\frac{{\left[ A \right]_{tot}}^{2}}{{K_{D}}^{2}}\left[ T \right]$. (5)

The log-linear range can be shifted up or down by changing [A]_tot_. Since the equation is linear over this range of target concentrations, this is equivalent to shifting the curve left or right:

$\log_{10} \left[ TA_{2} \right]=\log_{10} \left[ T \right]+\log_{10} \frac{{\left[ A \right]_{tot}}^{2}}{{K_{D}}^{2}}$ (6)

**Supplementary Note 2 | Predictive tuning**

We have generally observed that approximate predictions of reporter DNA output can be made independently of the target molecule. That is, despite the unpredictable effects of variables such as the number of available epitopes, the quaternary structure of the protein under assay conditions, and the average affinity of the polyclonal antibodies for their target, the same probe conditions will produce approximately the same DNA output response curves for different targets (**Figure 4**). Therefore, we were able to develop a heuristic that is only a function of (1) the concentration of the target and (2) the desired reporter DNA output concentration.

Our heuristic works very well for targets that are at concentrations below the average K_D_ of the antibodies (*i.e.*, the binding curve isn’t starting to saturate) and above the limit of detection of the assay. In this regime, the log-log response curves are almost completely linear and can be approximated by:

$log[DNA] = \alpha*log[T] + \beta*log[probe] + \gamma$ (7)

where [T] is typically the log center of the target’s physiological concentration range, [probe] is the concentration of each dAb, and [DNA] is the desired output reporter DNA copy number. Parameters $\alpha$, $\beta$, and $\gamma$ are scalar factors that we fit using *scipy.optimize* default parameters and empirical data from a few targets and probe concentrations. To estimate the probe concentration, we solve for [probe] in Eq. 7, which gives us this heuristic equation:

$log[probe] = \frac{log \left[ DNA \right] -\alpha*lo{g \left[ T \right]}^{*} -\gamma}{\beta}$ (8)

Based on this approach, we used empirical data from the GDF-15 tuning shown in **Figure 3a**, data from standard 250 pM dAb for all four targets shown in **Figure 3d**, and IL-6 tuning data to fit for the parameters in our heuristic ($\alpha$=0.85, $\beta$=1.46, and $\gamma$=-17.34). Data provided in the Supplementary Source Data File. With these parameters, we were able to quickly estimate appropriate probe concentrations for IL-1ra and CRP (without depletant) to be 21 pM and 0.809 pM, respectively. We also tuned the optimum IL-6 probe concentration to 372 pM. The resultant signals were within a two-fold range of the target output of 1 fM reporter DNA (**Figure 3e**). This confirms that our heuristic was successful in producing good estimates of probe concentration without the need for laborious tuning.

**Supplementary Note 3 | Hook effect and capture bead concentration**

It is prudent to mention the well-known ‘hook effect’, where high target concentrations lead to decreased signal^4^. It is accurately predicted by Eq. 3 (**Supplementary Note 1**) that target concentrations greatly exceeding [dAb] make it improbable that two detection probes will localize on the same target, resulting in a loss of signal. However, we do not observe this in our data. For low-abundance proteins, the target concentrations are far below [dAb] and therefore never in the hook effect regime. And for high-abundance targets, the maximum concentration is imposed by the cAb density—once the beads are saturated, any excess target is washed away—so as long as [cAb] < [dAb], we will not observe the hook effect for high abundance targets either. Nevertheless, this effect is still important to consider during assay design.

**Supplementary Table 1 | Targets in our four-plex panel.** For CRP, IL-1ra, and IL-6, concentration ranges were estimated from the Plasma Proteome Database, taking the highest and lowest reported values across both plasma and serum, and excluding concentration values taken from patients with severe disease. GDF-15 concentrations were estimated from various R&D ELISA kit manuals.

| **Analyte** | **Molecular Weight (kDa)** | **Clinical Implication** | **Approximate Concentration Range** |
| --- | --- | --- | --- |
| C-reactive protein (CRP) | 120 | Heart-related diseases, colon cancer, general systemic inflammation | ~1–36 nM |
| Growth/differentiation factor 15 (GDF-15) | 24.6 | Various diseases including cancer, diabetes, and cardiovascular-related diseases | ~5–120 pM |
| Interleukin 1 receptor agonist (IL-1ra) | 25 | Inflammatory diseases involving intestines, lungs, kidneys, and systemic infectious diseases | ~0.8–60 pM |
| Interleukin 6 (IL-6) | 21 | Many inflammatory and autoimmune diseases including Alzheimer’s Disease, cancer, rheumatoid arthritis, and major depressive disorder | Undetected – 480 fM |

**Supplementary Table 2 |** Assay conditions.

**Supplementary Table 3 |** Standards 1–6 were made with the four analyte concentrations shown in each row.

| Standard | [CRP] pM | [GDF-15] pM | [IL-1RA] pM | [IL-6] pM |
| --- | --- | --- | --- | --- |
| 1 | 121,500 | 486 | 364.5 | 1.823 |
| 2 | 40,500 | 162 | 121.5 | 0.608 |
| 3 | 13,500 | 54 | 40.5 | 0.204 |
| 4 | 4,500 | 18 | 13.5 | 0.068 |
| 5 | 1,500 | 6 | 4.5 | 0.023 |
| 6 | 500 | 2 | 1.5 | 0.008 |

**Supplementary Table 4 |** Human quantification by EVROS and Luminex. Source data are provided as a Source Data file.

|  |  |  |  |  | **EVROS** |  |  |  |  |
| --- | --- | --- | --- | --- | --- | --- | --- | --- | --- |
|  | **H1** |  |  | **H2** |  |  | **H3** |  |  |
|  | **Avg** | **std** | **n** | **Avg** | **std** | **n** | **Avg** | **std** | **n** |
| CRP | 16,179 | 2,482 | 3 | 73,045 | 38,720 | 3 | 16,384 | 3105 | 3 |
| GDF-15 | 42.57 | 8.10 | 3 | 78.85 | 9.27 | 3 | 46.93 | 4.00 | 3 |
| IL-1ra | 5.92 | 1.35 | 3 | 15.23 | 1.58 | 3 | 8.38 | 0.16 | 3 |
| IL-6 | 0.08 | 0.03 | 3 | 0.29 | 0.13 | 3 | 5.20 | 0.59 | 3 |

|  |  |  |  |  | **Luminex** |  |  |  |  |
| --- | --- | --- | --- | --- | --- | --- | --- | --- | --- |
|  | **H1** |  |  | **H2** |  |  | **H3** |  |  |
|  | **Avg** | **std** | **n** | **Avg** | **std** | **n** | **Avg** | **std** | **n** |
| CRP | 419,721 | 5,977 | 2 | 2,792,351 | 102,264 | 2 | 201,502 | 13,876 | 2 |
| GDF-15 | 14.37 | 0.85 | 2 | 19.16 | 1.70 | 2.00 | 10.48 | 0.42 | 2 |
| IL-1ra | 0.81 | 0.01 | 2 | 1.66 | 0.12 | 2.00 | 0.99 | 0.11 | 2 |
| IL-6 | n.d. |  | 2 | 0.14 | 0.03 | 2.00 | n.d. |  | 2 |

**Supplementary Table 5 |** Sequences used in work. Also provided in Source Data file.

**Supplementary References:**

1. Rosenberg-Hasson, Y., Hansmann, L., Liedtke, M., Herschmann, I. & Maecker, H. T. Effects of serum and plasma matrices on multiplex immunoassays. *Immunol Res* **58**, 224–233 (2014).

2. Darmanis, S. *et al.* ProteinSeq: High-Performance Proteomic Analyses by Proximity Ligation and Next Generation Sequencing. *PLoS ONE* **6**, e25583 (2011).

3. Nong, R. Y. *et al.* Solid-phase proximity ligation assays for individual or parallel protein analyses with readout via real-time PCR or sequencing. *Nature Protocols* **8**, 1234–1248 (2013).

4. Akın, L. *et al.* Hook Effect: A Pitfall Leading to Misdiagnosis of Hypoaldosteronism in an Infant with Pseudohypoaldosteronism. *Horm Res Paediatr* **74**, 72–75 (2010).
