# Supplementary material for: Extending the dynamic range of biomarker quantification through molecular equalization
Source: Nat Commun. 2023 Jul 13;14:4192. doi: 10.1038/s41467-023-39772-z (PMC10344875; doi:10.1038/s41467-023-39772-z)
Supplement: Supplementary file 3 — Reporting Summary [file 41467_2023_39772_MOESM3_ESM.pdf]

nature portfolio

Corresponding author(s): Tom Soh

Last updated by author(s): Jun 1, 2023

Reporting Summary

Nature Portfolio wishes to improve the reproducibility of the work that we publish. This form provides structure for consistency and transparency in reporting. For further information on Nature Portfolio policies, see our Editorial Policies and the Editorial Policy Checklist.

Statistics

For all statistical analyses, confirm that the following items are present in the figure legend, table legend, main text, or Methods section.

n/a	Confirmed	
☐	☒	The exact sample size (*n*) for each experimental group/condition, given as a discrete number and unit of measurement
☐	☒	A statement on whether measurements were taken from distinct samples or whether the same sample was measured repeatedly
☒	☐	The statistical test(s) used AND whether they are one- or two-sided<br>*Only common tests should be described solely by name; describe more complex techniques in the Methods section.*
☒	☐	A description of all covariates tested
☒	☐	A description of any assumptions or corrections, such as tests of normality and adjustment for multiple comparisons
☐	☒	A full description of the statistical parameters including central tendency (e.g. means) or other basic estimates (e.g. regression coefficient) AND variation (e.g. standard deviation) or associated estimates of uncertainty (e.g. confidence intervals)
☒	☐	For null hypothesis testing, the test statistic (e.g. *F*, *t*, *r*) with confidence intervals, effect sizes, degrees of freedom and *P* value noted<br>*Give P values as exact values whenever suitable.*
☒	☐	For Bayesian analysis, information on the choice of priors and Markov chain Monte Carlo settings
☒	☐	For hierarchical and complex designs, identification of the appropriate level for tests and full reporting of outcomes
☒	☐	Estimates of effect sizes (e.g. Cohen's *d*, Pearson's *r*), indicating how they were calculated

Our web collection on statistics for biologists contains articles on many of the points above.

Software and code

Policy information about availability of computer code

Data collection	Sequencing data was collected with Illuminas sequencing hub BaseSpace. FastQ files were generated using FASTQ Generation v1.1.0.<br>Sequencing reads were compiled and counted using fastaptamer_count v1 package.<br>qPCR data was collected with bio-rad CFX Manager.
Data analysis	All data was analyzed using custom code available on: https://github.com/newmanst/evros<br>Figure reproduction instructions and respective notebooks can be pushed to github upon request.

For manuscripts utilizing custom algorithms or software that are central to the research but not yet described in published literature, software must be made available to editors and reviewers. We strongly encourage code deposition in a community repository (e.g. GitHub). See the Nature Portfolio guidelines for submitting code & software for further information.

Data

Policy information about availability of data

All manuscripts must include a data availability statement. This statement should provide the following information, where applicable:

- Accession codes, unique identifiers, or web links for publicly available datasets
- A description of any restrictions on data availability
- For clinical datasets or third party data, please ensure that the statement adheres to our policy

The datasets analyzed during the current study are available on the freely accessible Stanford Data Repository: https://purl.stanford.edu/pf688zn8684

Human research participants

Policy information about studies involving human research participants and Sex and Gender in Research.

Reporting on sex and gender	N/A: HRP not applicable to this study. Only 3 human serum samples were used just to demonstrate measurement of endogenous analytes in human serum. However, the sex, number, and age of participants for the 3 samples used obtained from BioIVT are presented in the Methods.
Population characteristics	N/A
Recruitment	N/A
Ethics oversight	N/A

Note that full information on the approval of the study protocol must also be provided in the manuscript.

Field-specific reporting

Please select the one below that is the best fit for your research. If you are not sure, read the appropriate sections before making your selection.

☒ Life sciences ☐ Behavioural & social sciences ☐ Ecological, evolutionary & environmental sciences

For a reference copy of the document with all sections, see nature.com/documents/nr-reporting-summary-flat.pdf

Life sciences study design

All studies must disclose on these points even when the disclosure is negative.

Sample size	3 separate human serum samples were used to measure the analytes. This was chosen due to the limited throughput of Luminex processes.
Data exclusions	As stated in the text, two of the three human serum sample reads from Luminex were below the limit of detection, and thus could not be quantified.
Replication	Replicates were chosen to be 3 for everything except for the Luminex measurements. Due to the limited throughput and serum sample volume available, we had to limit Luminex replicates to be 2. All attempts at replication are presented in the figure plots.
Randomization	The three random human serum samples were obtained from BioIVT - random samples were provided to us.
Blinding	Sequence processing was blinded as samples were sent out to the Functional Genomics Facility for sequencing. All other data was not blinded because the samples were processed and analyzed by the same person due to limited personell. This is not very relevant to the experimental results as only comparisons within the same sample and the two measuring methods were done.

Reporting for specific materials, systems and methods

We require information from authors about some types of materials, experimental systems and methods used in many studies. Here, indicate whether each material, system or method listed is relevant to your study. If you are not sure if a list item applies to your research, read the appropriate section before selecting a response.

Materials & experimental systems

n/a	Involved in the study	
☐	☒	Antibodies
☒	☐	Eukaryotic cell lines
☒	☐	Palaeontology and archaeology
☒	☐	Animals and other organisms
☒	☐	Clinical data
☒	☐	Dual use research of concern

Methods

n/a	Involved in the study	
☒	☐	ChIP-seq
☒	☐	Flow cytometry
☒	☐	MRI-based neuroimaging

Antibodies

Antibodies used	Antibodies used were procured from R&D systems.<br>AF1707, AF206, AF280, AF957, AF4240, BAF1707, BAF206, BAF280

Validation

Validation statements are on the manufacturer website:
https://www.rndsystems.com/products/human-c-reactive-protein-crp-antibody_af1707
https://www.rndsystems.com/products/human-il-6-antibody_af-206-na
https://www.rndsystems.com/products/human-il-1ra-il-1f3-antibody_af-280-na
https://www.rndsystems.com/products/human-gdf-15-antibody_af957
https://www.rndsystems.com/products/gfp-antibody_af4240
https://www.rndsystems.com/products/human-c-reactive-protein-crp-biotinylated-antibody_baf1707
https://www.rndsystems.com/products/human-primate-il-6-biotinylated-antibody_baf206
https://www.rndsystems.com/products/human-il-1ra-il-1f3-biotinylated-antibody_baf280
Data are also provided as binding curves in the sequencing and qPCR data of the main text.
